# Individual differences in music-induced interpersonal synchronization and self–other integration: the role of creativity and empathy

**DOI:** 10.1098/rsos.240654

**Published:** 2024-11-13

**Authors:** Adrian Kempf, Pieter-Jan Maes, Canan Gener, Andrea Schiavio

**Affiliations:** ^1^ Department of Psychology, University of Graz, Glacisstraße 27, Graz 8010, Austria; ^2^ Institute for Psychoacoustics and Electronic Music, Ghent University, Miriam Makebaplein 1, Ghent B-9000, Belgium; ^3^ School of Arts and Creative Technologies, University of York, York YO10 5GB, UK

**Keywords:** interpersonal synchronization, self–other integration, creativity, music cognition, empathy, mixed-reality

## Abstract

It has been demonstrated that moving together in synchrony to music makes us feel connected. Yet, little is known about the individual differences that shape the relationship between interpersonal synchronization to music and social bonding. The present research tests the hypothesis that this association is influenced by differences in empathy and creativity–two highly relevant factors in many musical activities. We implemented a synchronization task featuring a virtual drummer and measured self–other integration (SOI), a core component of social bonding. We employed a dual-measurement paradigm, incorporating both an explicit assessment (*Inclusion of Other in the Self* scale) and an implicit assessment (*joint-Simon effect*) of SOI. Surprisingly, our analysis did not reveal explicit and implicit measurements correlating, nor were they similarly affected by interpersonal synchronization. This raises questions about the assessment of SOI in interpersonal synchronization experiments. Furthermore, we observed no moderating role of empathy or creativity in the association between interpersonal synchronization and SOI. Nevertheless, we found creativity to correlate with SOI. In light of this finding, we recommend placing greater emphasis on creativity as a decisive factor in the study of musical interaction.

## 1. Introduction

Music brings people together. Across many different cultural and social contexts, music involves a range of intersubjective activities such as singing in a choir, playing in a band, learning to play an instrument with a teacher or dancing with others. A wide spectrum of research has demonstrated that synchronized movement plays a key role in constituting shared musical experience as it helps establish social connections within and between audience members and musicians [1–3]. With this in mind, it has been argued that one of the most important roles that music serves in human evolution is facilitating social bonding, with synchronous movement being one of the key mechanisms to achieve this [4–7]. Recent research has also shown that an association between synchronization and social bonding can be found when interacting with virtual partners, such as computer-generated avatars [8–10].

**Figure 1.**
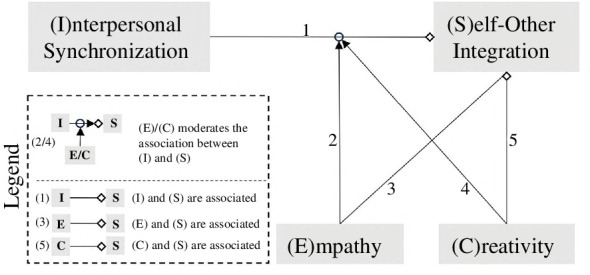
Proposed relationships between the variables of interest. This visualization depicts how we propose that empathy and creativity might moderate the relationship between interpersonal synchronization and self–other integration, based on the literature reviewed in the introduction. The figure also shows the correlations between self–other integration and the factors of interpersonal synchronization, empathy and creativity.

It has been argued that one of the mechanisms mediating the relationship between synchronization and social bonding is self–other integration (SOI) [11–15]. When we synchronize with others, indeed, a preliminary form of pro-sociality may be instantiated through the alignment of our and other’s movements [16, p. 199; 17,18], which are co-represented via the same neural encoding [19]. Because of this, a great variety of studies have examined how SOI in groups can be facilitated by synchronous movements (see Rennung & Göritz [18] and figure 1, *connection 1* for a visualization of this association), leaving relatively unexplored the topic of how individual differences can shape such an association. The present article aims to fill this gap and examines empirically how two specific individual factors, trait empathy and creativity, could shape the relationship between synchronization and SOI. In what follows, we delve into the pivotal roles of these two factors within this particular context.

Trait empathy has been identfied as a key factor that may moderate the relationship between interpersonal synchronization and SOI [20,21]. Multiple studies have shown that more empathic people display an increased tendency for SOI [22–25]. This association is depicted as *connection 3* in figure 1. Nevertheless, the evidence for a role of empathy in the association between synchronization and SOI remains sparse. Pecenka & Keller [26] found that in musical group activities, a self-report measure of perspective-taking associated with SOI [27] correlates with the musicians’ ability of temporal prediction in interpersonal synchronization. Furthermore, synchronization ability, empathy and the personality characteristic ‘agreeableness’ are positively correlated [28], with the latter being associated with SOI [29]. However, to our knowledge, only a single study observed that the degree to which synchronization and SOI are correlated is influenced by trait empathy [30]. This moderating effect of empathy is visualized as *connection 2* in figure 1.

Besides empathy, creativity can be understood as another highly relevant factor in the association between synchronization and SOI. Indeed, music may be seen as a creative phenomenon par excellence that has been explored in a number of ways [31,32]. Recent research, for example, has shown how interpersonal synchronization and coordination provide a foundation for collective creative endeavours such as musical improvisation [20,33–36]. We propose creativity to play a highly significant role in the association between synchronization and SOI in two different ways. (i) We expect that more creative people might exhibit an increased tendency for SOI (see *connection 5* in figure 1). Divergent creative thinking has been shown to promote SOI in a task involving synchronous movement [37]. Moreover, a strong correlation between SOI and the extraversion trait, associated with creativity [38], has been observed [29]. (ii) We also anticipate that more creative individuals might have an advantage in leveraging synchronization to increase SOI (see *connection 4* in figure 1). Increased SOI promotes cooperation among group members and could hence support collective creative performance [39–43].

With this in mind, the main goal of the present research is to investigate the twofold role of creativity and empathy in shaping the association between synchronization and SOI.

**RQ_1_**: What roles do empathy and creativity play in the interpersonal synchronization–SOI association? We expect that empathy and creativity both correlate with SOI and moderate the association between synchronization and SOI.

To address this question, we implemented a synchronization task with an avatar—a virtual drummer (VD)—to minimize any potential effects of social preferences that could emerge with a human partner. We contrasted conditions with and without synchronization to investigate the association between synchronization and SOI. Participants engaged in two variants of the synchronization task: one time they tapped in synchrony with a rigid VD, and the other time the VD dynamically adapted to the rhythm tapped by the participant. In the baseline condition without synchronization, participants just watched the VD play a drum beat. To measure SOI, we deployed an explicit and an implicit measurement. As an explicit measurement, we administered the commonly used ‘Inclusion of Other in the Self’ (IOS) scale [44,45]. For an implicit assessment of SOI, we measured the joint-Simon effect (JSE) through running a joint-Simon task (JST) [46]. The JSE has been shown to correlate with the IOS [47], but also with other variables linked to SOI, as for example perspective-taking [27]. Using the JSE in addition to the IOS scale allows us to back up our findings with an implicit measurement of SOI that it is not prone to expectancy effects. This might involve participants indicating a higher level of SOI as they expect synchronization to have such an effect. Such expectancy effects, it has been argued, may threaten the validity of research on the synchronization–SOI association [48]—but see Tunçgenç *et al*. [49] for a counter-argument. Due to our dual-measurement approach, we can also explore if the IOS scale is a valid explicit assessment method in studying the synchronization–SOI association with virtual partners. Indeed, as our study crucially depends on the assessment of SOI, we are equally interested in the validity of our proposed dual-measurement paradigm in assessing the association between synchronization and SOI.

**RQ_2_**: Do implicit (JSE) and explicit (IOS) measurements of self-other integration correlate? We anticipate that ratings on the IOS scale will correlate with the size of the JSE. Moreover, we expect the differences in JSE and IOS between conditions to be correlated among the two measurements.

We investigate the effect of synchronization on SOI using the dual-measurement paradigm in three different conditions.

**RQ_3_**: How does synchronization affect SOI with the VD? Based on previous studies [8,10], we expect SOI (as measured by the JSE and the IOS) to increase when participants synchronize with the VD in contrast to just watching the VD play. The use of an adaptive avatar in one of the conditions might further increase the amount of SOI.

## 2. Methods

### 2.1. Participants

We recruited 50 adult participants (14 males, 35 females and 1 other; age: *M* = 25.76, s.d. = 6.77). All participants were fluent in English and Dutch. Our sample size was guided by the effect size needed to address research question RQ_2_. This decision was made as our project resources were strictly limited and we expected RQ_2_ to require the largest sample size. The sample size was chosen using a simulation-based power analysis to detect the smallest effect size of interest (SESOI) of *r* ≥ 0.23 for a Spearman correlation with a power of 80% at an alpha level of 0.05. This SESOI was chosen based on the correlation between IOS and JSE reported by Shafaei *et al*. [47]. Consequently, as the sample size was chosen based on RQ_2_, the same effect size can be reliably detected when testing for the main effects in RQ_1_. Concerning the differences in SOI between conditions in RQ_3,_ our sample size should suffice as studies usually report medium to large effect sizes of *d* > 0.5 (e.g. [8,36]). Before taking part in the study, all participants gave informed consent.

### 2.2. Materials

We administered five questionnaires to assess participants’ musical training, empathy and general creativity. We measured musical training via the musical training subscale of the Goldsmiths Musical Sophistication Index (GMSI) [50]. Empathy was measured with the help of the brief Interpersonal Reactivity Index (B-IRI) [51], focusing on cognitive and affective aspects of empathy. Creativity was assessed using two different inventories: the short scale of creative self (SSCS) [52] and the assessment of everyday creativity across nine domains (AEC) [53]. Additionally, participants completed the ‘openness to experience’ subscale of the Big Five Inventory (BFI-O) [54].

Participants’ creativity was further assessed via the alternative use task (AUT) [55] and the remote associates task (RAT) [56], which are commonly used to measure divergent and convergent creative thinking ability, respectively. In the AUT, participants were presented with five everyday objects (i.e. candle, knife, sock, pencil and lamp), and were invited to come up with as many creative uses for each object as possible within two minutes. All responses to the AUT were rated by 10 independent judges for their creativity on a five-point Likert scale ranging from 1 (‘not at all creative’) to 5 (‘extremely creative’). The judges were asked to consider the novelty and appropriateness of the responses when rating their creativity. The inter-rater reliability across all everyday objects amounted to α = 0.61 (95% CI [0.58, 0.64]) as calculated using a Cronbach alpha test. The RAT was administered in Dutch and featured 22 items [57]. Each item comprised three words and the aim was to come up with a fourth word within 27 s that could be associated with the other words, e.g. room/blood/salts with the solution bath. The participant’s performance in the RAT task was scored by counting the number of correct responses [56].

**Figure 2.**
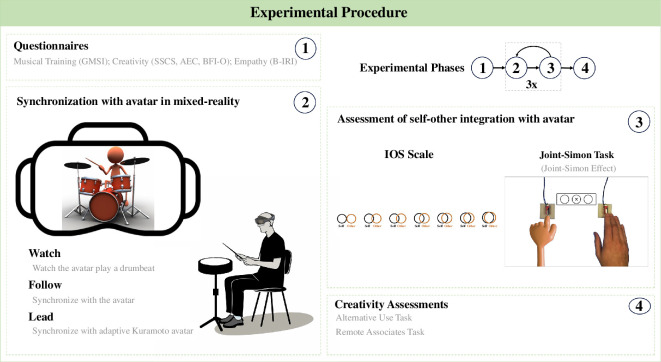
Experimental procedure. Visualization of the experimental procedure composed of four phases. (1) Participants were asked to complete multiple questionnaires. (2) Each participant ran through the synchronization task in three conditions, where they either watched the VD, or synchronized with them. (3) After each synchronization task, the participant’s SOI was measured on the IOS scale and via the JST. (4) At the end of the experiment, participants completed the AUT and RAT creativity assessments.

The main task of the experiment involved participants synchronizing with a three-dimensional virtual drummer (VD) playing a simple drumbeat at around 112 beats per minute (b.p.m.) (see figure 2 where an orange version of the avatar is depicted). We recorded the VD using full-body motion capture on a drummer with more than 10 years of experience. The drummer was asked to perform a simple and repetitive drum pattern in a 4/4-time signature using only the bass drum, snare and ride cymbal. The ride cymbal was played on every beat to clearly indicate the pulse with which the participant should synchronize. The drum pattern was recorded for 2 min, and a single 32-bar excerpt (approximately 69 s) was used in the experimental task, as this section had the highest motion capture quality. Although the drum pattern was recorded to a click track, there were some minor divergences from isochrony. We chose not to quantize the recording to retain the natural feel of the performance.

The VD was created in Unity 3D and visually presented to participants within a mixed-reality using the Microsoft HoloLens 2. The audio track was played to the participants using a spatial audio system aligned with the position of the VD in the room. Participants were invited to synchronize with the VD by drumming on an electronic drum pad to the beat with a drumstick. A similar movement was carried out by the VD, which was hitting a cymbal on every beat. The synchronization task featured three different conditions: in condition ‘Watch’ participants watched the VD; in condition ‘Follow’ participants synchronized by moving along with the VD, tapping the cymbal at around 112 b.p.m.; condition ‘Lead’ was similar to ‘Follow’ but involved an adaptive VD based on a Kuramoto algorithm [58,59]. Participants were not informed that the VD in condition ‘Lead’ would adapt to their movements. The synchronization task across all three conditions consistently used the same 32-bar drum pattern. Participants had two bars (8 beats) to listen to the drum pattern before they were required to tap along with the VD for 30 bars, totalling 120 taps. Thus, each trial of our synchronization task lasted approximately 64 s, which is between the 17 s trials used by Stupacher *et al.* [30] and the 3 min synchronization by Tarr *et al*. [10].

The automatic synchronization of the VD’s tapping to the real-time tapping of the participant in condition ‘Lead’ was realized using the Kuramoto coupled oscillator model by Maes *et al.* [60]. By considering the periodic tapping of both the participant and the VD as two oscillators (as modelled by equation (2.1)),^1^ the Kuramoto model enabled us to adjust the phase of the virtual drummer’s tapping over time towards in-phase synchronization with the participant’s tapping


(2.1) $$\frac{d\theta_{VD}}{dt}=\omega_{VD}+\frac{K}{2}\sin\left(\theta_{VD}-\theta_{P}\right)$$

The VD had a different colour in each condition to avoid potential carry-over effects: ‘Watch’/turquoise, ‘Follow’/orange, and ‘Lead’/purple. The experimenter always explicitly referred to the VD using their colour, e.g. the ‘orange drummer’’, to make clear that participants were not always interacting with the same VD.

Two methods were used to assess SOI with the VD. First, we administered an adapted version of the IOS scale to the participants [8,44,45]. We used the original question ‘Which picture best describes your relationship with the turquoise/orange/purple drummer (other)?’ But instead of using discrete Likert responses, SOI was rated on a continuous slider (value range from 1 to 7 in steps of 0.1). This rating slider was placed below the IOS scale. Second, participants carried out the JST together with the VD [46,61]. In the JST, participants were presented with a fixation cross in the centre of a screen. The participant’s hand was placed on a response button to the right of the fixation cross, while they could see the VD’s hand on a similar button to the left of the fixation cross. In each trial, a dot, either coloured green or yellow, was presented to the left or right of the fixation cross. Before the start of the task, participants were instructed to respond only to one of the two colours (go trials) while the VD responded to the other colour (no-go trials). As soon as the dot was presented, participants were asked to respond as fast as possible by pressing the response button if the dot in the instructed colour would appear, independent of its spatial location. The VD was animated to carry out the same task but responded to the dot of the other colour. The response of the VD consisted of a small movement of the index finger pressing the response button with a randomized response time between 266 ms and 466 ms, based on the study by Tsai & Brass [61] using a comparable JST with a virtual co-actor. The JSE can then be calculated by taking the difference between the average response time of the participant for spatially congruent (dot on the right) and incongruent (dot on the left) trials. The JSE has been shown to measure SOI [62] and to correlate with the assessment through the IOS scale [47].

### 2.3. Procedure

The experiment was carried out in the Art and Science Interaction Lab (ASIL) at Ghent University, Belgium, and took approximately one hour per participant. Every experimental session began with a practice phase in which participants could get accustomed to the main tasks by running through a single synchronization trial and one set of 20 JST trials (10 go and 10 no-go trials). This practice trial involved tapping along to the same 32-bar drum pattern that was also used during the experimental trials, but was only carried out with a non-adaptive VD. The VD had a neutral black colour during the practice trial in both the synchronization task and JST. All participants understood the instructions effortlessly and performed the tasks with ease.

Following the trial phase, participants completed a set of questionnaires (GMSI, B-IRI, SSCS, AEC and BFI-O). This was followed by the main phase of the experiment, in which participants ran through one trial of the synchronization task in each of the three conditions. The order of the conditions (‘Watch’, ‘Follow’ and ‘Lead’) was fully counterbalanced. On completion of the synchronization task in each condition, the participant’s SOI was assessed using the IOS scale and by carrying out the JST together with the VD. The colour of the VD during the JST was the same as in the prior condition of the synchronization task. The JST involved 50 go trials and 50 no-go trials, with half of each set of trials being spatially incongruent and the other half being spatially congruent trials. After participants ran through all three conditions of the synchronization task, they completed the AUT before the RAT. The procedure is visually summarized in figure 2.

This experiment has been pre-registered (https://aspredicted.org/R3P_QFC), including study design, hypotheses, sample size and statistical tests, unless otherwise stated.

### 2.4. Data analysis

We used the software ‘R’ [63] and ‘RStudio’ [64] for the data analysis. Linear mixed models were calculated with the package ‘lme4’ [65] and *p*-values for the coefficients of these models were computed using ‘lmerTest’ [66]. If factor variables were used as predictors, we applied suitable coding schemes as described by Schad *et al*. [67]. All post hoc comparisons for the linear mixed models were calculated using the package ‘emmeans’ [68] and in the case of multiple comparisons, *p*-values were adjusted using the Tukey method. Post hoc comparisons were not pre-registered, but used to simplify the presentation and interpretation of the linear (mixed) models. Where possible, we chose to report only post hoc comparisons in the results section instead of mixing them with regression coefficients. This decision was made because the regression coefficients often match the results of the post hoc comparisons due to the contrast coding of the regression coefficients. Complete model summaries are available in the electronic supplemental material, S1. Inferiority tests for Spearman correlations (estimated by linear models) were calculated using Fisher’s *z* transformation [69,70]. The analysis can be reproduced using the R script provided in our online repository linked in the electronic supplementary material.

For the analysis of the recorded reaction times in the JST, we excluded any trials with reaction times that diverged more than 2.5× s.d. from the condition mean [71]. Participants were excluded from the analysis of the JST if more than 30% of their trials were either excluded or incorrect, i.e. not giving a response on a go trial [47]. In total, 1183 (approx. 8%) of 15 000 trials were excluded from the analysis. This exclusion criterion has not been pre-registered, but is a common procedure in studies using the JST (see [71–73]).

Furthermore, we pre-registered to exclude participants if they did not achieve a stable synchronization with the VD in condition ‘Lead’. As we only recorded the phase difference between the participants’ and the VD’s tapping signal, we did not detect outliers based on the correlation of the tapping signals. However, we ran a Rayleigh test using the ‘circular’ [74] package in ‘R’, which tests against a uniformity of phase differences between two signals. The test was significant (*p* < 0.001) for all participants in conditions ‘Follow’ and ‘Lead’ indicating a unimodal distribution of phase differences between the participants and the VD. Hence, all participants achieved synchrony during the tapping trials [75] and no participant needed to be excluded from the analysis.

To analyse our participants’ creativity, we constructed a composite variable ‘Creativity’ by averaging across the scores of all creativity self-assessment questionnaires (SSCS, AEC and BFI-O). While all three questionnaires cover different aspects of creativity, averaging can be justified by all three being interrelated, as shown both in the literature and in our study [52,76–78]. The SSCS measures creative self-efficacy and creative personal identity and correlates positively with creativity and divergent thinking tests [52]. The BFI-O is a personality measurement test that is strongly associated with divergent thinking [76], but also creativity ability [77] and everyday creative activities [78]. The latter is measured by the AEC questionnaire by asking participants how often they engage in different tasks associated with everyday creativity. In our study, high reliability was found across all items of these three questionnaires with a Cronbach alpha value of α = 0.86, 95% CI [0.8, 0.91].

Surprisingly, our composite variable ‘Creativity’ for the self-assessment creativity questionnaires did not significantly correlate with the AUT or RAT score (*r*_AUT × Creativity_ = 0.04, 95% CI [−0.31, 0.37], *p* ≥ 0.999; *r*_RAT × Creativity_ = −0.09; 95% CI [−0.42, 0.26], *p* ≥ 0.999). A possible explanation is the participants’ poor performance in the AUT and RAT tasks. Both tasks were situated at the end of the experiment, where participants might have already been fatigued. In the RAT, participants on average only gave *M* = 2.79 (s.d. = 2.49) correct responses for 22 items. Similarly, the mean fluency across items in the AUT (*M* = 6.31, s.d. = 4.13) was well below the average observed in creativity studies (*M* = 9.08, 95% CI [7.54, 10.61] for 114 items from 31 studies) [79]. In addition to participant fatigue, our choice of AUT items might have contributed to the low fluency scores. Therefore, we decided to conduct separate analyses for the composite variable ‘Creativity,’ which was constructed from the self-assessment questionnaires, as well as for the AUT, and RAT scores.

## 3. Results

An overview of all models used in our analysis can be found in table 1. A complete summary of each model is included in the electronic supplementary material S1. In what follows, we delve into the main results of the study as follows: we begin by addressing RQ_3_, exploring the association between synchronization and SOI by reporting the outcomes of models 1, 2 and 3. We then focus on our RQ_2_ and examine the correlation between explicit and implicit measurements of SOI via models 4, 5 and 6. Finally, we return to our RQ_1_, and explore the role of empathy and creativity in the interpersonal synchronization–SOI association with the remaining models 7–14.

**Table 1.** Summary of all linear (mixed) models used in the data analysis. The models’ formula is stated in the lme4 syntax. ‘*x:y*’ signifies the interaction between two variables *x* and *y* as a predictor; ‘x*y’ signifies the variables *x* and *y* as a predictor as well as their interaction; ‘(1|*z*)’ signifies a varying intercept for variable *z*; a variable ‘*x*’ that has been rank-transformed is listed as rank(*x*). The reaction time (in ms) recorded in the JST is abbreviated as RT (ms), the mean phase divergence of the participant’s tapping signal in comparison to the VD is abbreviated as *M*(φ)_divergence_.

model	outcome	formula			factor levels				contrasts
1	IOS	condition + (1|subject)			condition (‘Watch’, ‘Follow’, ‘Lead’)				sliding differences
2	RT (ms)	condition*spatial_congruency + (1|Subject)			condition (‘Watch’, ‘Follow’, ‘Lead’), spatial_congruency (‘true’, ‘false’)				treatment
3	M(φ)_divergence_	condition + (1|subject)			condition (‘Follow’, ‘Lead’)				sum
4	rank (JSE)	rank (IOS)			NA				sum
5	rank (JSE)	condition:rank (IOS)			condition (‘Watch’, ‘Follow’, ‘Lead)				sum
6	JSE	difference:IOS +(1|subject)			difference (‘Lead’–‘Watch’, ‘Follow’–Watch’, ‘Lead’–‘Follow’)				sum
7/8	IOS/JSE	condition*creativity + (1|subject)			condition (‘Watch’, ‘Follow’, ‘Lead’)				sum
9/10	IOS/JSE	condition*score_AUT_ + (1|subject)			condition (‘Watch’, ‘Follow’, ‘Lead’)				sum
11/12	IOS/JSE	condition*score_RAT_ + (1|subject)			condition (‘Watch’, ‘Follow’, ‘Lead’)				sum
13/14	IOS/JSE	condition*empathy + (1|subject)			condition (‘Watch’, ‘Follow’, ‘Lead’)				sum
**model**	**σ^2^**	**τ_00_ subject**	**ICC**	**subjects**		**observations**	**marginal *R* ^2^**	**conditional *R* ^2^**	
1	1.04	1.36	0.57	50		150	0.101	0.611	
2	2565.19	846.81	0.25	49		6317	0.007	0.253	
3	0.02	0.00	0.03	50		100	0.075	0.102	
4	NA	NA	NA	NA		141	<0.001	NA	
5	NA	NA	NA	NA		141	0.016	NA	
6	1578.95	324.47	0.17	50		150	0.013	0.182	
7	1.00	1.39	0.58	50		150	0.117	0.630	
8	283.95	49.68	0.15	49		141	0.098	0.233	
9	1.04	1.42	0.58	48		144	0.114	0.625	
10	283.44	73.92	0.21	47		137	0.056	0.251	
11	1.06	1.38	0.57	50		150	0.102	0.610	
12	282.62	68.99	0.20	49		141	0.052	0.238	
13	1.05	1.37	0.57	50		150	0.105	0.611	
14	286.06	70.34	0.20	49		141	0.040	0.229	

σ2 (residual variance); τ00 (between-group variance); ICC (intraclass correlation coefficient); marginal *R* ^2^ (explained variance by fixed effects); conditional *R* ^2^ (explained variance by fixed and random effects).

### 3.1. Association between synchronization and SOI (RQ3)

First, we examined whether music-induced synchronization increases SOI with the VD—a finding that has been reported in previous research [8–10,30].

#### 3.1.1. Model 1/Differences in IOS between conditions

**Figure 3.**
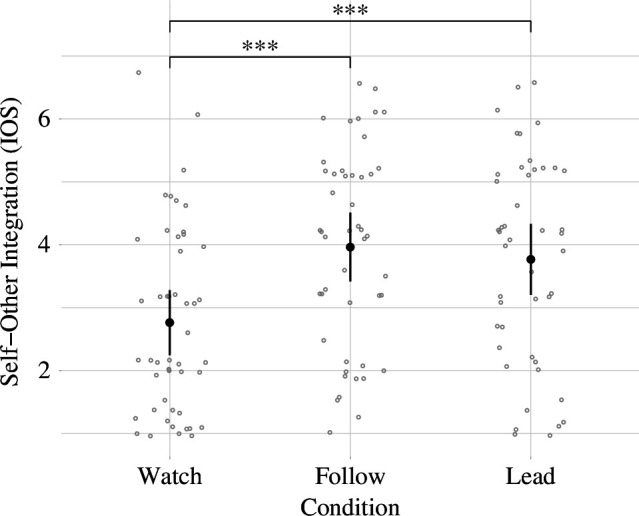
Visualization of the mean SOI as measured on the IOS scale for every condition with 95% confidence intervals, corrected as proposed by Morey [80]. The figure also includes the individual measurements of SOI for every participant within each condition. (*** / *p* < 0.001)

We constructed model 1 with IOS as an outcome variable, condition as a predictor and a varying intercept for subject. Post hoc comparisons showed that the IOS significantly increased (‘Follow’–‘Watch’: M_Follow–Watch_ = 1.2, *t*(98) = 4.65, *p* < 0.001, *d* = 1.18, 95% CI_d_ [0.75, 1.61]; ‘Lead’–‘Watch’: M_Lead–Watch_ = 0.95, *t*(98) = 5.89, *p *< 0.001, *d* = 0.93, 95% CI_d_ [0.51, 1.35]) in conditions ‘Follow’ and ‘Lead’ compared to the condition ‘Watch’. No significant difference (‘Follow’–‘Lead’: M_Follow–Lead_ = 0.25, *t*(98) = 1.24, *p* = 0.434, *d* = 0.25, 95% CI_d_ [−0.15, 0.65]) in IOS was found between the conditions ‘Follow’ and ‘Lead’ (see figure 3).

#### 3.1.2. Model 2/Differences in JSE between conditions

**Figure 4.**
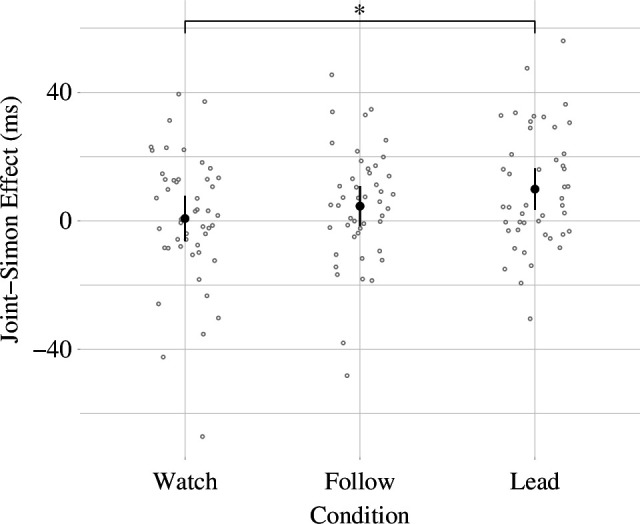
Visualization of the mean SOI as measured by the JSE for every condition with 95% confidence intervals, corrected as proposed by Morey [80]. The figure also includes the individual measurements of the JSE for every participant within each condition. (* / *p* < 0.05)

For the second measurement of SOI through the JSE, we built model 2 with the reaction times in the JST as an outcome variable and a varying intercept for the subject. The variable condition as well as the spatial congruency of stimulus and response (a factor variable with two levels: true/false) and their interaction were included as predictors. The variable condition and spatial congruency were both coded using treatment contrasts. Post hoc comparisons showed a significant (JSE_Follow_: M_JSE_ = 6.02 ms, *t*(6263) = 2.7, *p* = 0.007, *d* = 0.12, 95% CI_d_ [0.03, 0.21]; JSE_Lead_: M_JSE_ = 9.66 ms, *t*(6263) = 4.38, *p* < 0.001, *d* = 0.19, 95% CI_d_ [0.11, 0.28]) JSE for conditions ‘Follow’ and ‘Lead’, while no significant (JSE_Watch_: M_JSE_ = 2.21 ms, *t*(6263) = 1.01, *p* = 0.314, *d* = 0.04, 95% CI_d_ [−0.04, 0.13]) JSE was found for condition ‘Watch’. We observed the JSE to be significantly (JSE_Lead–Watch_: ß = 0.13, 95% CI [0.02, 0.23], *p* = 0.017) increased in condition ‘Lead’ compared to condition ‘Watch’ (see figure 4). No significant difference (JSE_Follow–Watch_: ß = 0.07, 95% CI [−0.04, 0.17], *p* = 0.223) in JSE was found between conditions ‘Follow’ and ‘Watch’.

#### 3.1.3. Model 3/Differences in synchronization accuracy between conditions

We also compared the synchronization accuracy in conditions ‘Follow’ and ‘Lead’. While this test was only pre-registered as an exploratory analysis, it helps to understand the difference between conditions ‘Follow’ and ‘Lead’ with respect to participants’ synchronization performance. To assess the synchronization accuracy, we calculated the mean phase divergence M(φ)_divergence_. The φ_divergence_ is equal to the phase difference between the VD and the participant in case the tap of the latter is rushed. Otherwise, if the participant is dragging, the φ_divergence_ is equal to the phase difference minus 2π. The mean phase divergence M(φ)_divergence_ was included as outcome variable in model 3. The model was further composed of condition (a factor with two levels: ‘Follow’ and ‘Lead’) as predictor and a varying intercept for the subject. The predictor condition was coded using sum contrasts. We observed a significant reduction (ß = −0.60, 95% CI [−0.97, −0.22], *p* = 0.002) of synchronization accuracy in the condition ‘Lead’ compared to the condition ‘Follow’.

### 3.2. Correlation between explicit and implicit measurements of SOI (RQ2)

We then examined whether our explicit measurement of SOI (IOS) correlates with our implicit measurement of SOI (JSE), as suggested by Shafaei *et al*. [47].

#### 3.2.1. Model 4/Correlation between IOS and JSE across conditions

We constructed model 4 to calculate the correlation between IOS and JSE across conditions. Model 4 included the average JSE per participant as the outcome variable and the IOS as the predictor. The variables JSE and IOS were both rank transformed using the rank function in ‘R’. No significant (ß < 0.001, 95% CI [−0.17, 0.17], *p* = 0.992) correlation was found between IOS and JSE. An inferiority test was conducted against the null hypothesis that the correlation is greater than or equal to the SESOI of *r* = 0.23. Using Fisher’s *z* transformation yielded a value of *z* = −2.74 (95% CI[−2.91,−2.57]) showing that the observed correlation is significantly smaller (*p* = 0.003) than the SESOI.

#### 3.2.2. Model 5/Correlation between IOS and JSE within conditions

We also computed model 5 to look at the correlation between IOS and JSE within each condition. Model 5 consisted of the average JSE per participant as the outcome variable and the interaction of condition and IOS as the predictor. The variables JSE and IOS were both rank transformed. No condition displayed a significant (ß_Watch_ = 0.13, 95% CI[−0.17, 0.43], *p* = 0.396; ß_Follow_ = −0.14, 95% CI[−0.43, 0.16], *p* = 0.367; ß_Lead_ = 0.005, 95% CI[−0.29, 0.30], *p* = 0.974) correlation between IOS and JSE. Inferiority tests against the null hypothesis that the correlation is greater than or equal to the SESOI were performed for the correlations in all three conditions. For conditions ‘Watch’ and ‘Follow’, Fisher’s *z* transformation showed that the observed correlations are significantly smaller (*z*_Watch_ = −2.28, 95% CI[−2.57,−1.98], *p*_Watch_ = 0.011; *z*_Follow_ = −1.79, 95% CI[−2.10, −1.50], *p*_Follow_ = 0.036) than the SESOI. In contrast, we could not reject the null hypothesis we tested against with the inferiority test for condition ‘Lead’ as indicated by the results of Fisher’s *z* transformation (*z*_Lead_ = −1.33, 95% CI[−1.43,−1.33], *p*_Lead_ = 0.128).

#### 3.2.3. Model 6/Correlation of differences in IOS and JSE between conditions

Model 6 was computed to test if the differences in IOS and JSE between conditions are correlated among these two measurements. The model included the average JSE per participant as the outcome variable and the interaction of a factor coding each difference between conditions with the IOS. The difference in IOS and JSE between conditions was not found to be significantly correlated between any two conditions (ß_Lead–Watch_ = 0.03, 95% CI[−0.15, 0.21], *p* = 0.739; ß_Follow–Watch_ = -0.08, 95% CI[−0.26, 0.09], *p* = 0.357; ß_Lead–Follow_ = −0.09, 95% CI[−0.36, 0.18], *p* = 0.521).

### 3.3. The role of empathy and creativity in the interpersonal synchronization and self–other integration association (RQ1)

Furthermore, we were interested in how different measurements of creativity and empathy are associated with SOI as assessed by the IOS and the JSE.

#### 3.3.1. Models 7 and 8/Association of the composite variable ‘Creativity’ with the IOS and JSE

**Figure 5.**
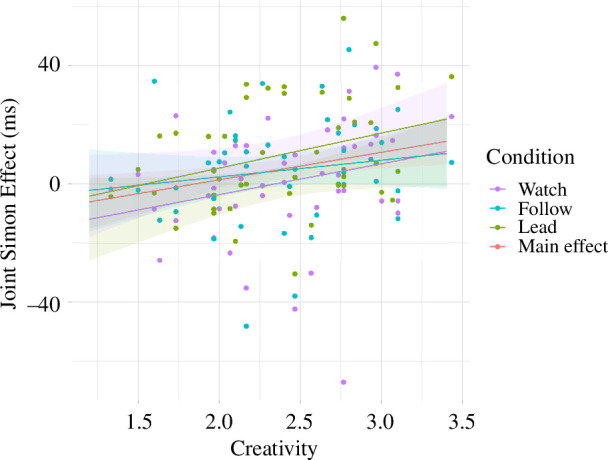
Visualization of the correlations between the composite variable ‘Creativity’ and the JSE. The correlation is depicted for the significant main effect across all conditions and within every condition including the 95% confidence intervals. The correlation within the conditions is only significant for the condition ‘Lead’. The figure also shows the variable ‘Creativity’ in combination with the JSE for every participant in each condition.

We computed model 7 with IOS and model 8 with the JSE as the outcome variable. Both models were composed of conditions (coded using sum contrasts), the variable ‘Creativity’ and their interaction as predictors and a varying intercept for the subject. For model 7, we did not find a significant correlation (ß = −0.07, 95% CI[−0.30, 0.16], *p* = 0.543) between ‘Creativity’ and the IOS. Furthermore, we did not find ‘Creativity’ to significantly moderate the association between synchronization and IOS (‘Follow’–‘Watch’: M_IOS~Creativity*(Follow–Watch)_ = −0.88, *t*(96) = −2.11, *p* = 0.092, *d* = −0.88, 95% CI_d_ [−1.90, 0.14]; ‘Lead’–‘Watch’: M_IOS~Creativity*(Lead-Watch)_ = −0.75, *t*(96) = −1.81, *p* = 0.173, *d* = −0.75, 95% CI_d_ [−1.77, 0.27]; ‘Follow’ – Lead’: M_IOS~Creativity*(Follow–Lead)_ = −0.129, *t*(96) = −0.31, *p* = 0.949, *d* = −0.13, 95% CI_d_ [−1.14, 0.89]). Looking at model 8, we found a significant correlation (ß = 0.24, 95% CI [0.06, 0.42], *p* = 0.011) between ‘Creativity’ and the JSE. Post hoc comparisons showed that the correlation between ‘Creativity’ and the JSE is only significant in condition ‘Lead’ (M_Lead_ = 11.82, 95% CI [0.87, 22.8], *d* = 0.62, 95% CI_d_ [0.02, 1.20], *p* = 0.035) and not significant in conditions ‘Watch’ and ‘Follow’ (M_Watch_ = 10.43, 95% CI [−0.32, 21.2], *p* = 0.057; M_Follow_ = 5.66, 95% CI [−5.23, 16.5], *p* = 0.316). The correlation between ‘Creativity’ and the JSE is depicted in figure 5 as the main effect as well as for every condition. We did not find ‘Creativity’ to moderate the association between synchronization and JSE (‘Follow’–‘Watch’: M_JSE~Creativity*(Follow–Watch)_ = −4.77, *t*(90) = 0.67, *p* = 0.783, *d* = −0.28, 95% CI_d_[−1.32, 0.75]; ‘Lead’–‘Watch’: M_JSE~Creativity*(Lead–Watch)_ = 1.38, *t*(90.3) = 0.193, *p* = 0.980, *d* = 0.08, 95% CI_d_[−0.96, 1.12]; ‘Follow’ – ‘Lead’: M_ISE~Creativity*(Follow–Lead)_ = −6.16, *t*(91) = −0.85, *p* = 0.672, *d* = −0.37, 95% CI_d_[−1.41, 0.68]).

#### 3.3.2. Models 9 and 10/Association of the AUT score with the IOS and JSE

Two models similar to models 7 and 8 were calculated but using the average AUT score as the predictor. Models 9 and 10 included the IOS and JSE, respectively, as the outcome variables. We did not find any relevant significant effects for models 9 and 10.

#### 3.3.3. Models 11 and 12/Association of the RAT score with the IOS and JSE

Similar models to models 7 and 8 were calculated using the RAT score as the predictor rather than ‘Creativity’. Models 11 and 12 included the IOS and JSE, respectively, as the outcome variables. We did not find any relevant significant effects for models 11 and 12.

#### 3.3.4. Models 13 and 14/Association of empathy with the IOS and JSE

Finally, we were interested in whether empathy moderates the effect of synchronization on SOI. We constructed model 13 with IOS and model 14 with the JSE as the outcome variables. Both models included empathy (as measured by the B-IRI questionnaire) and condition (coded using sum contrasts) as predictors, as well as a varying intercept for participants. We did not observe a significant main effect of empathy on IOS and JSE (ß_IOS~empathy_ = 0.08, 95% CI [−0.15, 0.30], *p* = 0.503; ß_JSE~empathy_ = 0.03, 95% CI [−0.17, 0.22], *p* = 0.796). Moreover, for neither measurement of SOI did we find empathy to play a significantly moderating role in the association between synchronization and SOI (‘Follow’–‘Watch’: M_IOS~empathy*(Follow–Watch)_ = 0.11, *t*(96) = 0.19, *p* = 0.99, *d* = 0.11, 95% CI_d_ [−1.00, 1.21]/ M_JSE~empathy*(Follow–Watch)_ = −0.84, *t*(89.3) = −0.09, *p* = 0.995, *d* = −0.05, 95% CI_d_ [−1.17, 1.07]; ‘Lead’–‘Watch’: M_IOS~empathy*(Lead–Watch)_ = 0.258, *t*(96) = 0.451, *p* = 0.894, *d* = 0.25, 95% CI_d_ [−0.85, 1.36]/ M_JSE~empathy*(Lead–Watch)_ = −1.44, *t*(89.7) = −0.15, *p* = 0.988, *d* = 0.08, 95% CI_d_ [−1.21, 1.04]; ‘Lead’–‘Follow’: M_IOS~empathy*(Lead–Follow)_ = 0.15, *t*(96) = 0.29, *p* = 0.964, *d* = 0.14, 95% CI_d_ [−0.96, 1.25] / M_JSE~empathy*(Lead–Follow)_ = −0.6, *t*(89) = −0.06, *p* = 0.998, *d* = 0.04, 95% CI_d_ [−1.16, 1.09]).

In all, the study’s main findings reveal several key insights. First, neither empathy nor creativity played a significant moderating role in the association between synchronization and SOI. However, creativity did show a correlation with SOI, as measured by the JSE. Interestingly, we found no correlation between the explicit measurement of SOI (IOS) and the implicit measurement (JSE). Moreover, differences in SOI between conditions did not correlate across the IOS and JSE measurements. Specifically, SOI, as measured by the IOS scale, significantly increased when participants synchronized with the VD, compared to when they only watched the VD. Additionally, the JSE, serving as an implicit measurement of SOI, showed a significant increase in condition ‘Lead’ when participants synchronized with the adaptive avatar compared to condition ‘Watch’. The discrepancy between the explicit and implicit measurements of SOI highlights the complexity of these interactions and the need for further investigation into their underlying mechanisms. In §4, we discuss these findings and frame them relative to existing scholarship, suggesting avenues for future research and theory.

## 4. Discussion and conclusion

We examined if, and to what extent, individual differences in creativity and empathy influence the association between interpersonal synchronization and SOI in a mixed-reality musical setting. We invited our participants to synchronize with a VD that was either adaptive or not. In doing so, we investigated the validity of the IOS scale—the most common explicit assessment method of SOI—when synchronizing with virtual others. For that reason, we deployed a dual-measurement approach to assessing SOI by also including an implicit measurement determining the JSE via the JST. We obtained several interesting findings concerning our main research questions:

1. Which role do empathy and creativity play in the interpersonal synchronization–SOI association? Neither empathy nor creativity were found to moderate the effect of synchronization on SOI. However, we observed creativity to be correlated with SOI, as measured by the JSE.
2. Do implicit (JSE) and explicit (IOS) measurements of SOI correlate? We did not observe a correlation between the explicit and implicit measurement of SOI. Across all conditions and within conditions ‘Watch’ and ‘Follow’, the association between both measurements was significantly smaller than previously reported [47]. We also did not find the differences in IOS and JSE between conditions to be correlated among the two measurements.
3. How does interpersonal synchronization affect SOI with the VD? SOI as measured on the IOS scale was significantly increased when participants synchronized with the VD, when compared to the condition where participants only watched the VD. The JSE as implicit measurement was only significantly increased in condition ‘Lead’ (in comparison to condition ‘Watch’) when participants synchronized with the adaptive avatar.

In what follows, we will first discuss the results concerning questions (II) and (III) because the investigation of the role of empathy and creativity in the association between synchronization and SOI decisively depends on the assessment of SOI. Both implicit (JSE) and explicit (IOS) measurements need to reliably assess the effect of interpersonal synchrony on SOI in every condition. As we have observed significant differences between the implicit (JSE) and explicit (IOS) measurements of SOI in all conditions, these findings need to be scrutinized first, before elaborating on our main question (I).

### 4.1. Explicit and implicit measurements of self–other integration

We did not observe a correlation between the explicit (IOS) and implicit (JSE) measurement of SOI as previously reported by Shafaei *et al*. [47]. Moreover, these two measurements also differ with respect to conditions and predictors: first, in contrast to the IOS, the JSE is not significantly increased in the synchronization condition ‘Follow’ when compared to the condition ‘Watch’. Additionally, the differences in IOS and JSE between conditions were not correlated for any pair of conditions. Second, concerning predictors of SOI, the correlation between measurements of creativity and JSE was not observed for the IOS.

Our primary intention in deploying a dual-measurement paradigm was to back up our findings on the IOS scale using an implicit measurement, as it has been suggested that explicit measurements of SOI are prone to expectancy effects [48]. In the study by Atwood *et al*. [48], the authors asked participants to imagine a synchronization experiment, and indicate their expectations about the closeness of the people synchronizing. Indeed, it was found that participants’ expectations matched the outcomes usually reported in synchronization experiments. Hence, explicit measurements of SOI might reflect participants’ expectations about the effect of synchronization rather than the amount of SOI. Yet, Atwood *et al*.’s study [48] has been criticized for possibly not considering all empirical evidence, and for potentially being affected by demand characteristics itself [49].

A detailed inspection of our findings shows that the results of our study do not fully support Atwood *et al*.’s argument [48], that the effect of synchronization on SOI relies solely on participants’ expectations. Similar to the IOS, we did find the implicit measurement through the JSE to be increased in condition ‘Lead’ in contrast to ‘Watch’. And while we did not find this increase in JSE for condition ‘Follow’, we found a significant JSE in both synchronization conditions. Both results indicate that synchronization affects SOI. Furthermore, considering our analysis of the correlation between IOS and JSE, we merely point out that the association is significantly smaller than expected based on previous results [47].

There are multiple reasons why the dynamics of JSE and IOS between conditions do not align, and the correlation is smaller than expected. First, our experiment only examines synchronization with virtual co-actors, limiting our results and critique to this paradigm. The rigidity of virtual co-actors and the general context of interacting within a virtual environment might explain why the correlation between IOS and JSE is smaller than expected. For example, we did not find the correlation of IOS and JSE to be significantly smaller than expected in the ‘Lead’ condition, where participants interacted with an adaptive avatar. This suggests that the correlation of IOS and JSE might be stronger with more realistic co-actors.

Second, the IOS scale and the JSE might measure two different aspects of SOI. The JSE, on the one hand, assesses SOI on a bodily (minimal self) level [14,81]. When performing the JST, we arguably co-represent the actions of the other person involved, such as the VD reacting to the stimulus by pressing the button. The co-actors’ actions are represented as action–effect bindings, which overlap with the action–effect bindings for our own actions [19]. This results in an increased level of SOI. On the other hand, the IOS scale was developed to measure SOI on a narrative self-level with a focus on romantic relationships [82]. In such relationships, people integrate and relate to others’ perspectives and resources as if they were their own when they speak and think about themselves. Yet, these two different aspects of SOI might be related [81]. Research has, for instance, demonstrated that romantic partners show increased JSE compared to people in non-romantic relationships [14]. Furthermore, a correlation between JSE and IOS has been shown for couples in various relationship forms, i.e. friends, colleagues and partners [47]. In contrast, our findings suggest that when synchronizing music with virtual others, the relationship between the two aspects of SOI may be limited.

Third, it is possible that the effect of synchronization on SOI, as measured by the IOS scale, may still be influenced by participants’ expectations. This could account for some of the discrepancies between the explicit and implicit measurements. Moving forward, while the JST provides an implicit measure, future studies should focus on disentangling demand characteristics from other confounding variables, such as the choice of assessment method or the experimental setting (e.g. online, virtual or in-person dyads).

In all, our findings highlight the importance of distinguishing between different aspects of SOI, and the need to develop and validate more reliable assessment methods. While the IOS scale provides a convenient way to assess SOI compared to implicit methods like the JSE, it may not be suitable for research focused on the bodily level of SOI. Particularly in studies involving synchronization with virtual others, it is crucial to determine the strength of the correlation between IOS and JSE to assess whether the association is practically relevant, especially if the correlation is very small.

### 4.2. The effect of interpersonal synchronization on SOI

Compared to the ‘Watch’ condition, we found that IOS scores were higher in the ‘Follow’ and ‘Lead’ conditions when participants synchronized with the VD. However, the JSE was significantly increased only in the ‘Lead’ condition compared to the baseline condition ‘Watch’. The increase of the IOS through synchronization replicates previous findings of the association between interpersonal synchronization and SOI when interacting with virtual others [8–10,30]. Against our expectations, participants did not show a higher IOS in condition ‘Lead’, where the avatar adapted to the participant when compared to condition ‘Follow’. We assumed that an adaptive avatar would Lead to higher SOI due to a decrease in the mismatch between the participants’ and the VD’s rhythmic movements which made the interaction more predictable for the participant [83]. As we noted that the IOS may capture different aspects of SOI than the JSE and could be influenced by expectancy effects, our result is difficult to interpret. However, turning to our findings for the JSE, they support our hypothesis as we found the JSE to be significantly increased in condition ‘Lead’ compared to both other conditions.

This still leaves the question open as to why the JSE is only significantly increased (compared to condition ‘Watch) in one of the synchronization conditions, namely ‘Lead’. As mentioned above, during the JST, the actions of our co-actor are represented via action–effect bindings, also called events, which overlap with the action–effect bindings we use to represent our own actions [19]. According to the referential coding account, the size of the JSE depends on the degree of event similarity [84]. Hence, the more similar one’s own action becomes to the actions of a co-actor, the more difficult it gets to distinguish between the cognitive representations of one’s own and others’ action–effect bindings. Fairhurst *et al*. [85] already have shown that synchronizing with an overly adaptive avatar leads to the activation of the precuneus—a brain region involved in discriminating between one’s own and other’s action–effect representations, which is often necessary for experiencing agency and perspective-taking [86]. The effect of synchronization on SOI as measured by the JSE might therefore be especially pronounced when interacting with a strongly adaptive avatar, as movements of the participant and the avatar are more aligned.

Our analysis of synchronization accuracy clearly shows that participants’ movements are more aligned with the VD’s movements in condition ‘Lead’ than ‘Follow’. This could explain why we found the JSE in condition ‘Lead’ to be significantly different from condition ‘Watch’, while we did not find such an effect for condition ‘Follow’. As the VD in condition ‘Follow’ is completely rigid and does not adapt at all to the participant, the degree of similarity is likely lower than in condition ‘Lead’. Importantly, we still found a significant JSE in condition ‘Follow’ as we also did in condition ‘Lead’. This shows that the degree of similarity, while probably not as high as in condition ‘Lead’, was pronounced enough to lead to a JSE. We assume that one might observe a JSE in between the measurements of condition ‘Lead’ and ‘Follow’ with a real human synchronization partner where phases of following and leading flexibly alternate.

### 4.3. The role of empathy and creativity in the association between interpersonal synchronization and self–other integration

Our third main finding can be summarized as follows: empathy and creativity do not moderate the association between synchronization and SOI. However, only creativity, not empathy, is correlated with SOI. Similarly to the study by Stupacher *et al*. [30], we expected empathy to correlate with SOI and play a moderating role in the association between synchronization and SOI. However, it could be that our sample size did not suffice to measure the previously reported role of empathy. Moreover, against our hypothesis, people with higher creativity were not more capable of capitalizing on synchronization to increase SOI. Yet, we observed that more creative people had higher levels of SOI as measured by the JSE. We should point out that we have found the latter result only for the assessment of creativity through questionnaires such as the ‘Assessment of Everyday Creativity’, ‘Short Scale of Creative Self’ and the ‘Big Five Inventory: Openness to Experience’ which were combined into a compound measurement.

Our findings can be seen to align with those reported by Zabelina *et al*. [87]. The authors observed participants with high levels of creative achievement to show ‘leaky’ attention: in other words, the higher their level of creative achievement the more they were influenced by information in the environment as reflected in the response times of a reaction time task. While we did not directly measure *creative achievement*, our compound measurement of creativity incorporates assessments of creative self-concept, openness to experience and a report of creative activities, which are all predictors of creative achievement [52,78,88–91]. Thus, participants with higher scores on our compound creativity measurement could also have been prone to leaky attention and be more sensitive to task information in the JST. Consequently, they might have had more complex co-representations of their co-actors, including details such as movement dynamics and timings or specific aspects of the environmental context. All of this could be reflected in the JSE, as discriminating between one’s own and the other’s event representations gets more difficult the more similarities are represented.

A further similarity to the findings by Zabelina *et al*. [87] is that in our study the potential effect of leaky attention was only indicated by the relationship between response times (in our case the JSE) and the compound measurement of creativity. We did not observe such an association for the assessments of divergent or convergent creative thinking (AUT and RAT, respectively). Comparably, Zabelina *et al*. [87] did not find a relationship between divergent thinking ability and response times. These parallels aside, future research is needed to corroborate this interpretation of our results by directly assessing creative achievement, for example by using the ‘Creative Achievement Questionnaire’ [92].

### 4.4. Limitations and future directions

Several limitations of our study have already been pointed out. While we determined our sample size based on a previous finding of the correlation between IOS and JSE [47], a larger sample size would have been helpful in determining the actual effect size in the scenario of synchronizing with a virtual other. An increase in sample size might also be necessary to replicate the finding by Stupacher *et al*. [30] on the role of empathy in the synchronization–SOI association. Their study, with a sample size of 162, had more than three times the number of participants compared to our study. Moreover, as we only used a non-adaptive and a strongly adaptive avatar, future work might profit from using virtual co-actors with different levels of adaptivity [93] or a varying predictability of movements [94]. Such an increase in so-called ‘behavioural realism’ might be especially important compared to a more realistic appearance of the co-actor, as has been shown by research investigating the interaction with virtual agents [95]. A further step might be the use of interaction tasks that lead to synchronized behaviour without explicitly manipulating it, e.g. through musical improvisation. Nonetheless, our study marks a starting point in critically assessing the measurement of SOI in synchronization experiments with virtual others.

## 5. Conclusion

We have provided a plausible explanation for the positive correlation observed between creativity and the extent of SOI. This explanation is based on the observation that individuals with high levels of creative achievement show leaky attention. However, this finding raises the question of whether people with higher levels of creativity indirectly benefit more from synchronization. While we did not find them to directly profit from synchronization to a greater extent than others, they might benefit from a higher level of SOI compared to people with lower levels of creativity. Indeed, these increased levels of SOI might help them to act cooperatively [41]. Yet, while willingness to cooperate is a necessary prerequisite for collective endeavours, successful creative collaboration likely also depends on other factors such as, for example, flexibly switching between working collectively and individually [96]. Future research might also deploy similar dual measurements in synchronization experiments involving non-virtual co-actors to investigate the different levels of SOI involved in interpersonal synchronization to music. Finally, our finding that creativity correlates with SOI in the context of interpersonal musical synchronization is further evidence for the need to consider creativity as a major factor in studying music cognition.

## Data Availability

Data and code used in this study are available at [97]. Supplementary material is available online [98].

## Footnotes

^1^

Equation (2.1): The Kuramoto model as it is applied to two oscillators; the periodic tapping of the participant ( $\theta_{VD}$ = phase of the rhythm) and the VD ( $\theta_{VD}$ = phase of the rhythm, $\omega_{VD}$ = angular velocity and $\theta_{VD}$ = the required rate of phase change to achieve synchrony with the participant). The parameter *K* defines the coupling strength. For the experiment, the coupling strength *K* was set to 2, leading to rapid adjustments by the VD to the participant’s input taps, resulting in strong automatic synchronization.
